# Beyond Description: A Functional GIUS-Based Algorithm for Enteral Feeding Decisions in the ICU

**DOI:** 10.3390/jpm16070376

**Published:** 2026-07-14

**Authors:** Nick Weidner, Christian von Loeffelholz, Robert Patejdl, Jan Seyfferth, Heinrich Volker Groesdonk

**Affiliations:** 1 Department of Intensive Care, Helios Clinic Erfurt, 99089 Erfurt, Germany; heinrich.groesdonk@helios-gesundheit.de; 2 Department of Anaesthesiology and Intensive Care, Jena University Hospital, Friedrich Schiller University, 07743 Jena, Germany; christian.von_loeffelholz@med.uni-jena.de; 3 Department of Physiology, Health and Medical University, 99089 Erfurt, Germany; robert.patejdl@hmu-erfurt.de; 4 Department of Gastroenterology, Helios Clinic, 99089 Erfurt, Germany; jan.seyfferth@helios-gesundheit.de

**Keywords:** GIUS, enteral nutrition, feeding decision

## Abstract

Gastrointestinal dysfunction is common in critically ill patients and frequently compromises the delivery and tolerance of enteral nutrition. Traditional bedside markers such as gastric residual volume or nonspecific abdominal symptoms provide only limited diagnostic accuracy and often fail to capture dynamic alterations in gastrointestinal function. Gastrointestinal ultrasound (GIUS) has emerged as a noninvasive, bedside-applicable method that enables structural and functional assessment of the gastrointestinal tract and may support more individualized nutritional management in intensive care. This narrative review summarizes the physiology and pathophysiology of gastric emptying and intestinal transit in critically ill patients, reviews established GIUS protocols, including Gastrointestinal and Urinary Tract Sonography (GUTS), Acute Gastrointestinal Injury Ultrasound Scoring (AGIUS), the Lai protocol, and the Ultrasound Meal Accommodation Test (UMAT), and proposes pragmatic GIUS-based algorithms for enteral feeding decisions. Three clinical use cases are addressed: 8 h monitoring during ongoing enteral nutrition, preprandial assessment of feeding readiness, and once-daily screening of gastrointestinal function. Current evidence supports the clinical relevance of key sonographic parameters such as gastric antral cross-sectional area and small-bowel diameter, whereas other measures, including mucosal thickness, colonic wall thickness, and Doppler-derived resistive indices, require further validation. UMAT adds a dynamic component to static sonographic assessment and may improve the evaluation of gastric accommodation and emptying in selected patients. Structured GIUS protocols offer a promising, evidence-informed extension of bedside assessment for enteral nutrition management in the intensive care unit. However, the available literature remains heterogeneous and is largely based on physiological studies, observational cohorts, and expert consensus. Prospective multicenter studies are needed to validate cutoff values, training standards, and outcome effects before GIUS-based algorithms can be adopted as stand-alone decision tools.

## 1. Introduction

Monitoring gastrointestinal function remains one of the central challenges in modern intensive care medicine [1,2,3]. Disorders of the stomach, small intestine, and colon are common in critically ill patients and strongly influence clinical outcomes [1]. Traditional clinical markers—such as gastric residual volume, abdominal symptoms, or intra-abdominal pressure—are limited in their diagnostic accuracy and often fail to adequately capture dynamic changes [4]. To address these limitations and to provide this important clinical field with greater visibility and a more robust scientific framework, a Delphi consensus process was recently conducted. Its results further supported the integration of imaging-based approaches into gastrointestinal function monitoring [5]. Gastrointestinal ultrasound (GIUS) has emerged as a novel, non-invasive, bedside-appropriate method that enables a differentiated assessment of gastrointestinal function by linking structural and functional information, and it is increasingly being integrated into intensive care treatment algorithms [1,6].

GIUS allows direct visualization of abdominal organs and their dynamic processes. It facilitates the detection of motility disorders, dilatation, wall abnormalities, and pathological fluid collections while enabling a more objective assessment of enteral feeding tolerance and longitudinal monitoring [1]. Beyond improving the characterization of gastrointestinal dysfunction, GIUS-based protocols have been associated with established scores of gastrointestinal failure, feeding intolerance, and short-term mortality in critically ill patients, underlining the potential clinical relevance of GIUS-guided monitoring for patient outcomes [7]. In parallel, the Ultrasound Meal Accommodation Test (UMAT) has been developed as a dynamic functional tool that quantifies gastric accommodation and emptying after food intake [8,9].

The aim of this narrative review is to outline the physiological and pathophysiological mechanisms of intra-enteric nutrient transport in relation to ultrasound-based assessment. Building on this foundation, we propose pragmatic approaches that may serve as practical recommendations for monitoring enteral nutrition. To this end, established protocols such as Gastrointestinal and Urinary Tract Sonography (GUTS), Acute Gastrointestinal Injury Ultrasound Scoring (AGIUS), and the Lai protocol are integrated with the Ultrasound Meal Accommodation Test (UMAT) into a structured, clinically oriented framework [7,10,11].

First, we summarize the relevant gastrointestinal physiology and its clinical implications. We then review established gastrointestinal ultrasound protocols, including UMAT. Finally, we propose pragmatic GIUS-based algorithms for enteral feeding decisions and discuss their application using a representative case example in Appendix A. The proposed algorithms should be understood as a pragmatic, hypothesis-generating framework derived from the available literature and clinical reasoning.

## 2. Materials and Methods

This work was designed as a structured narrative review addressing the use of gastrointestinal ultrasound for the assessment of gastrointestinal function and enteral feeding tolerance in critically ill adults. The review was conducted and reported with reference to the principles of the Scale for the Assessment of Narrative Review Articles (SANRA).

A structured literature search was performed in PubMed from database inception to March 2026. Search terms combined concepts related to “gastrointestinal ultrasound”, “gastric ultrasound”, “intestinal ultrasound”, “gastric emptying”, “feeding intolerance”, “enteral nutrition”, “critical illness”, “intensive care”, “GUTS”, “AGIUS”, “Lai protocol”, and “Ultrasound Meal Accommodation Test”.

Studies were eligible if they included adult critically ill patients and reported gastrointestinal ultrasound parameters, protocol-based GIUS assessment, enteral feeding tolerance, gastrointestinal dysfunction, or clinically applicable reference values. Physiological and perioperative studies were retained only when they provided relevant information regarding measurement methodology or the derivation of sonographic reference values. These studies were explicitly distinguished from ICU-specific evidence. Pediatric studies, animal studies, publications without relevant sonographic data, and reports lacking sufficient methodological detail were excluded.

Titles and abstracts were screened by 3 reviewers, followed by full-text assessment of potentially relevant publications. Data were extracted on study design, population, clinical setting, patient position, ultrasound technique, investigated parameter, proposed cutoff value, reference standard, and clinical endpoint.

Because of substantial heterogeneity in study populations, ultrasound techniques, patient positioning, definitions of feeding intolerance, and reported outcomes, no quantitative meta-analysis was performed. Evidence was therefore synthesized narratively and categorized according to study design and clinical context. Proposed cutoff values were interpreted as study-derived reference values rather than universally validated treatment thresholds.

This article remains a structured narrative review and a hypothesis-generating clinical framework. It does not claim the completeness or evidentiary certainty of a systematic review, and the proposed GIUS framework is intended to supplement rather than replace established clinical assessment and guideline-based nutrition practice.

Use of Generative Artificial Intelligence (GenAI): Generative artificial intelligence (ChatGPT, OpenAI, San Francisco, CA, USA; GPT-5.5) was used exclusively to support language editing, improve readability, and refine the structure of the manuscript. All scientific content, literature selection, interpretation of evidence, and final editorial decisions were performed independently by the authors, who reviewed and verified all generated text prior to submission.

## 3. Physiology of Gastric Emptying and Intestinal Transit

Gastric emptying is a highly complex, multiparametric process. Its reservoir function is essential for the rhythmic delivery of chyme into the small intestine, influencing not only nutrient absorption but also tolerance of enteral feeding—particularly in the intensive care setting [12,13,14,15].

### 3.1. Anatomical and Physiological Basics

#### 3.1.1. Anatomy and Functional Segments

**Figure 1:**
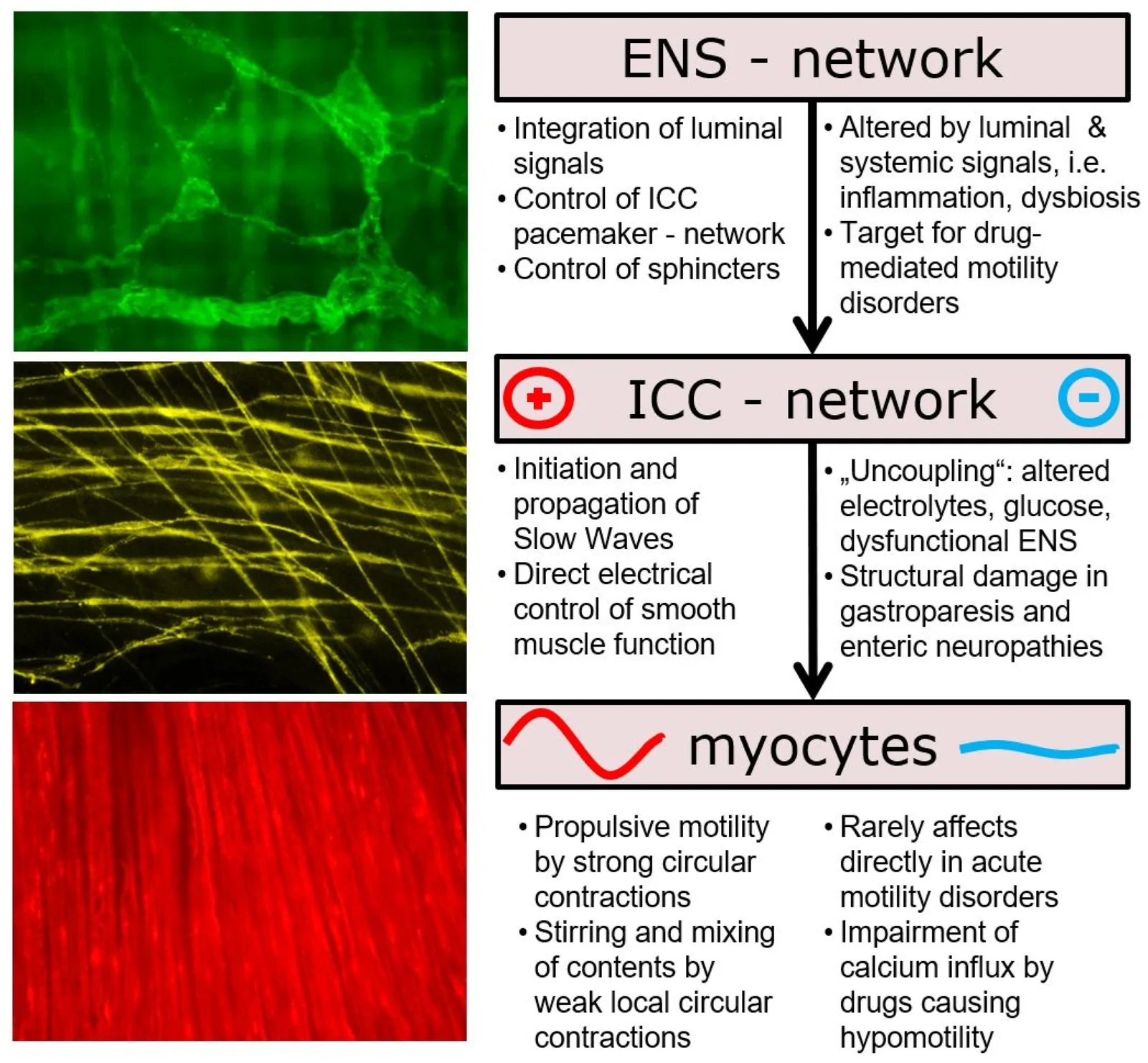
The complex motor patterns of the gastrointestinal tract are generated within a multicellular network of smooth muscle cells, interstitial cells of Cajal (ICC), and the highly complex neuronal networks of the enteric nervous system (ENS). Continuous bidirectional communication with luminal contents, microbiota, the autonomic nervous system, endocrine pathways, and immune cells governs gastrointestinal motility. This network is physiologically altered during aging and acute critical illness [20,21] The red (+) and blue (−) symbols denote excitatory and inhibitory influences, respectively. The red and blue waveforms schematically represent enhanced and reduced myocyte activity.

Morphologically, the stomach is divided into fundus, corpus, antrum, and pylorus. Following food intake, the fundus provides storage, the antrum provides mechanical trituration (“triturative function”), and the pylorus regulates outflow into the duodenum. Only finely triturated and sufficiently liquefied particles can pass through the narrow pyloric channel into the small intestine [14,16] (Figure 1).

#### 3.1.2. Neural Control

Neural regulation relies on the interaction of multiple systems. The enteric nervous system (ENS), consisting of the myenteric and submucosal plexuses, locally coordinates motility through excitatory (acetylcholine, substance P) and inhibitory (NO, VIP) signaling [17]. Sensory neurons detect stretch and chemical changes, initiating reflex arcs. Parasympathetic input—primarily via the vagus nerve—stimulates motility, while sympathetic activation suppresses it. Central control centers such as the nucleus tractus solitarius (NTS) and the dorsal motor nucleus of the vagus (DMV) modulate motility based on peripheral and central input and are integral components of physiological reflex circuits [18,19] Figure 1.

#### 3.1.3. Hormonal Regulation

Hormonal control reflects a balance between motility-promoting and inhibitory substances. During fasting, ghrelin and motilin stimulate antral contractions and initiate the migrating motor complex. Postprandially, inhibitory mediators predominate—including cholecystokinin (CCK), glucagon-like peptide-1 (GLP-1), peptide YY (PYY), and gastric inhibitory peptide (GIP)—thereby delaying gastric emptying and protecting the small intestine from overload [22,23].

#### 3.1.4. Mechanical and Chemical Factors

The rate of gastric emptying correlates with the physical and chemical properties of gastric contents. Fat-rich and hyperosmolar meals slow emptying, whereas isotonic, carbohydrate-rich liquids pass more rapidly. Particle size determines whether ingested material can leave the stomach. Gastric distension and wall stretch modulate neural and hormonal reflexes. Medications—particularly opioids and anticholinergics—reduce gastric emptying, whereas prokinetic agents may accelerate it. In addition, the widespread use of GLP-1 receptor agonists introduces new challenges because these agents can markedly delay gastric emptying and may induce drug-related gastroparesis in some patients [12,24,25,26].

#### 3.1.5. Physiology of Small Intestine and Colon

After entering the small intestine, chyme undergoes coordinated propulsion, mixing, and nutrient absorption. Motility is characterized by the migrating motor complex (MMC) during fasting [27] and by segmental and propulsive contractions after meals, regulated by enteric and vagal afferents as well as hormonal mediators such as secretin, CCK, and motilin. In critically ill patients, hypomotility, abnormal contraction patterns, and impaired absorptive capacity are common and may be driven by inflammation, medications, or metabolic dysregulation [28].

The colon serves as a site for absorption of water and electrolytes and the final processing of non-absorbed material. Its motility is characterized by slower, mass-propulsive movements and local segmental mixing [29]. Regulation is primarily enteric, with hormonal modulation and microbiota influence. In critically ill patients, motility is frequently disturbed due to paralytic ileus, toxic megacolon, or colorectal organ failure [30].

#### 3.1.6. Clinical Relevance in Critical Illness

Disorders of gastric emptying and gastrointestinal motility are frequent, multifactorial phenomena in critically ill patients and are of substantial clinical relevance. Based on the physiological control mechanisms described above—the finely tuned interaction between neural, hormonal, and mechanical regulation—severe illness gives rise to specific pathophysiological patterns that directly shape clinical management [3,5,28,31].

In the intensive care setting, external modulation through analgosedation, catecholamine therapy, and systemic inflammatory processes leads to dysfunction of the enteric nervous system, resulting in inhibition of normal motility reflexes and predominance of inhibitory sympathetic input [32,33]. In parallel, central vagal control circuits appear to be impaired, disturbing synchronization between the corpus, antrum, and pylorus. At the same time, critical illness and therapeutic interventions alter the secretion and activity of gastrointestinal hormones: postprandial regulation is dominated by inhibitory mediators such as cholecystokinin, glucagon-like peptide-1, and peptide YY, whereas endogenous stimulation by prokinetic peptides such as motilin and ghrelin is blunted [31,34,35].

These pathophysiological alterations manifest as delayed or absent antral contractility, persistent pyloric opening or tonic closure, and reduced duodenal propulsive force [36,37]. Mechanical and chemical factors—including altered nutrient composition of artificial nutrition, increased osmolality, local edema, or microischemia of the gastric wall—may further amplify these functional disturbances [32].

Clinically, these processes often present as marked gastroparesis with prolonged gastric retention and accumulation of gastric contents. Patients often demonstrate high gastric residual volumes, gastric dilatation, and an increased risk of regurgitation and aspiration-associated pulmonary complications [32,36]. In the small intestine, disturbed motility may result in hypomotility and diffuse dilatation and may promote bacterial overgrowth up to translocation of microbes into the systemic circulation [38].

Therefore, feeding tolerance in critically ill patients is significantly reduced. The inability to achieve adequate enteral nutrition leads to caloric deficits, fosters a catabolic metabolic state, prolongs ICU stay, and correlates with increased morbidity and mortality [39]. Current nutrition guidelines and scoring systems explicitly acknowledge these dysfunctions under the concept of gastrointestinal failure [40].

Against this background, a pathophysiological understanding of gastrointestinal function in critical illness is essential. It forms the basis for targeted functional diagnostics (e.g., GIUS and the Ultrasound Meal Accommodation Test [UMAT]), enables individualized nutritional management, and facilitates early recognition and treatment of potentially life-threatening complications [3,8,41,42,43].

### 3.2. Classical GIUS Protocols in Critical Care Medicine

#### 3.2.1. Role of GIUS

Over recent years, gastrointestinal ultrasound (GIUS) has become a central tool for the structural and functional assessment of the gastrointestinal tract in critically ill patients [1]. In contrast to clinical indicators or laboratory parameters, GIUS provides immediate, visual, dynamic, and quantitatively assessable information regarding anatomical integrity, motility, and—depending on the protocol—even perfusion of gastrointestinal segments. The use of structured protocols supports reproducibility and comparability of findings across the interdisciplinary ICU setting [11,44].

#### 3.2.2. Standardized Assessment of the Gastric Antrum

The gastric antrum is visualized in a sagittal view extending from epigastrium to a slightly right-sided plane. The left lobe of the liver serves as a landmark, whereas the aorta, the inferior caval vein and superior mesenteric vessels may be identified in close proximity. Measurements should be obtained during antral relaxation between peristaltic waves.

The anteroposterior (AP) and craniocaudal (CC) diameters are measured from serosa to serosa.

$$CSA=\frac{anteroposterior\;diameter\times craniocaudal\;diameter\times \pi }{4}$$

Patient position substantially influences antral dimensions and must therefore be reported together with the measured CSA. Serial measurements should be performed in the same position and imaging plane. For routine application of the proposed ICU algorithm, the supine or semi-recumbent position is used as the operational standard because it is feasible in most critically ill patients. Additional assessment in the right lateral decubitus position may improve the detection and quantification of gastric content when clinically feasible. However, thresholds obtained in the supine and right lateral decubitus positions should not be used interchangeably.

#### 3.2.3. The GUTS Protocol: Concept, Workflow, and Clinical Significance

The Gastrointestinal and Urinary Tract Sonography (GUTS) protocol is presented in Table 1. GUTS is a structured approach for systematic sonographic evaluation of the entire gastrointestinal tract in critically ill patients [10]. As described by Perez-Calatayud et al. [10], its focus is on a stepwise examination with clearly defined imaging windows, measurement points, and diagnostic criteria. The overarching aim is objective documentation and comparability of pathological findings, thereby supporting therapeutic decision-making.

By condensing findings into a structured assessment, the GUTS protocol facilitates longitudinal monitoring and interdisciplinary communication within the ICU team. The application and interpretation of defined cutoff values allow rapid, targeted therapeutic action, particularly in the assessment of complex gastrointestinal complications such as ileus, megacolon, intra-abdominal sepsis, or ischemia. The integration of perfusion diagnostics adds an additional dimension and may support early detection and monitoring of clinically relevant circulatory disturbances.

Although the protocol requires a high degree of sonographic expertise, its clear structure, algorithmic workflow, and threshold logic support reproducibility and clinical applicability. Bedside portability further facilitates its use, which is why GUTS is frequently regarded as a reference protocol for routine ICU practice and clinical research. However, accurate performance and interpretation still require substantial operator training and experience.

The authors advocate routine daily use; however, with an estimated time requirement of 10–15 min to complete all measurements, feasibility may be limited in increasingly demanding clinical environments. In addition, artifacts may hamper assessment and further highlight the practical limitations of the protocol.

#### 3.2.4. The AGIUS Protocol: Concept, Workflow, and Clinical Significance

The Acute Gastrointestinal Injury Ultrasound Score (AGIUS) protocol is a standardized scheme for the sonographic assessment of acute gastrointestinal dysfunction in critically ill patients. It was developed to transform ultrasound findings into a score-based evaluation system, thereby enabling more objective comparison and longitudinal monitoring, particularly in the intensive care context [1,3].

The AGIUS protocol is shown in Table 2. AGIUS comprises a systematic sonographic evaluation of the major gastrointestinal segments.

Each parameter evaluated by AGIUS is translated into a semiquantitative score reflecting the severity of acute gastrointestinal injury or dysfunction. The AGIUS score thereby facilitates longitudinal monitoring and objectifies dynamic clinical changes under therapy, such as during enteral nutrition, prokinetic treatment, or withdrawal of medications associated with gastrointestinal side effects. Importantly, its discriminative performance has been demonstrated by ROC analysis, with an area under the curve (AUC) of 0.827 for the prediction of feeding intolerance during the first week of ICU stay. Using a cutoff value of ≥3.5, the score achieved a sensitivity of 87.7% and a specificity of 82.4%, underscoring its diagnostic accuracy in this context. Studies have shown that higher AGIUS scores are associated with an increased risk of feeding intolerance, systemic complications, and worse outcomes such as prolonged mechanical ventilation or increased mortality [45,46,47,48]. A major advantage lies in straightforward documentation and monitoring, which allows objective comparisons between patients, serial examinations, and treatment phases.

Limitations include operator dependency, the need for ultrasound expertise, and—in daily routine—the time required to perform the complete scoring. Nevertheless, the AGIUS score is increasingly used as a structured tool for differentiated assessment of gastrointestinal function in the ICU.

#### 3.2.5. The Lai Protocol: Concept, Workflow, and Clinical Significance

The Lai protocol represents a focused, composite gastrointestinal ultrasound approach developed to support decisions regarding the initiation of enteral nutrition in critically ill patients with acute gastrointestinal injury. Rather than providing a comprehensive examination of all gastrointestinal segments, the approach combines selected gastric and colonic parameters to characterize gastric filling, colonic distension, and colonic motility [49].

The original retrospective single-center study included 105 adult intensive care patients with acute gastrointestinal injury grade II and an NRS-2002 score of at least 3. The sonographic examination focused on three principal parameters (Table 3).

- Stomach

The gastric antrum was visualized and its cross-sectional area (CSA) was determined by tracing the antral contour using Simpson’s integral method. Whenever clinically feasible, the examination was performed in the right lateral decubitus position. If lateral positioning was not possible because of the patient’s critical condition, measurements were obtained in the supine position. In both positions, the head of the bed was elevated by approximately 30–45°.

- Colon

The internal diameter of either the left descending colon or the right ascending colon was measured. The reported diameter therefore refers specifically to the colon and should not be interpreted as a small-bowel diameter threshold.

- Colonic motility

Colonic peristaltic activity was quantified by counting the number of contractions per minute in the examined colonic segment.

The first examination was performed before the initiation of enteral nutrition. Follow-up examinations were conducted after 24, 72, and 120 h, allowing the investigators to evaluate changes in gastrointestinal function during the initiation and advancement of enteral feeding. Enteral nutrition was commenced after clinical stabilization and reduction of vasoactive support, initially as trophic feeding and subsequently advanced in the absence of clinically relevant feeding intolerance.

This serial workflow differentiates the Lai protocol from purely descriptive gastrointestinal ultrasound examinations. Its principal objective is not the comprehensive anatomical assessment of the gastrointestinal tract but the integration of selected sonographic markers into a clinically applicable evaluation of readiness for enteral nutrition.

##### Clinical Significance

The Lai protocol, summarized in Table 4, combines gastric antral CSA, colonic diameter, and colonic peristaltic frequency to support the functional assessment of the upper and lower gastrointestinal tract. In the original study, a gastric antral CSA of ≤9 cm^2^, a colonic diameter of ≤2.9 cm, and a colonic peristaltic frequency of >3 contractions/min were associated with successful initiation and advancement of enteral nutrition.

The combined assessment demonstrated greater discriminative performance than the individual parameters and achieved an area under the receiver operating characteristic curve of 0.95. These findings support the concept that an integrated evaluation of gastric filling and colonic function may be more clinically informative than reliance on a single sonographic measurement.

However, the proposed values should be interpreted within the context of the original study. All three thresholds were derived from the same retrospective single-center cohort of patients with acute gastrointestinal injury grade II and have not yet been externally validated. Furthermore, the gastric antral CSA was primarily obtained in the right lateral decubitus position, while supine measurements were permitted when lateral positioning was not clinically feasible. As results were not reported separately according to patient position, the value of 9 cm^2^ should not be regarded as a universally applicable supine or right lateral decubitus cutoff.

The colonic diameter threshold refers specifically to measurements of the left descending or right ascending colon and is not applicable to the small intestine. In addition, the investigated clinical context was the initiation and early advancement of enteral nutrition rather than the diagnosis of feeding intolerance throughout the entire ICU stay.

Accordingly, the Lai protocol provides an important conceptual and evidentiary basis for multidomain GIUS assessment. Its thresholds are best regarded as study-derived reference values that support an integrated clinical decision rather than as independently validated criteria for automatically initiating, advancing, reducing, or withholding enteral nutrition.

### 3.3. Clinical Relevance of Sonographic Protocols for Nutritional Therapy—In Light of the Recommendations by Reintam Blaser et al. [1]

The structured and reproducible acquisition of gastrointestinal findings through standardized ultrasound protocols such as AGIUS, GUTS, and Lai represents a relevant advance in the guidance of enteral nutrition in critically ill patients. In contrast to the traditional reliance on gastric residual volume (GRV)—whose diagnostic validity and prognostic value have increasingly been questioned—these protocols allow integration of objective, therapeutically relevant parameters directly into clinical decision-making.

The publication by Reintam Blaser et al. [1] marked an important shift in the intensive care management of gastrointestinal function. The authors advocated a pragmatic approach and highlighted essential ultrasound parameters such as antral cross-sectional area, small- and large-bowel motility, detection of free intra-abdominal fluid, and assessment of wall abnormalities as decision aids for nutritional therapy. Their recommendations support moving beyond the limitations of traditional surrogate markers such as GRV and toward modern imaging-based diagnostics.

The three protocols operationalize these recommendations in different ways:

- The AGIUS protocol provides a score-based framework that semiquantitatively captures the severity of gastrointestinal dysfunction and enables objective longitudinal monitoring.
- The GUTS protocol combines a structured, algorithm-driven examination with integration of perfusion assessment and defined pathological cutoffs, thereby offering a basis for early detection and monitoring of complex complications.
- The Lai protocol standardizes measurement sites and techniques, thereby supporting reproducibility and comparability in both daily practice and clinical research.

In clinical practice, these protocols may facilitate earlier recognition of feeding intolerance, more targeted adaptation of therapeutic strategies, and prevention of complications such as gastroparesis, ileus, or aspiration pneumonia. The systematic integration of core sonographic parameters into therapeutic algorithms may also support rapid, needs-based, interdisciplinary management and is broadly aligned with current scientific recommendations.

In summary, the current evidence and consensus articulated by Reintam Blaser et al. [1] support the targeted use of pragmatic, multifactorial GIUS protocols as a promising framework for guiding enteral nutrition and preventing gastrointestinal complications in critically ill patients.

### 3.4. Dynamic Ultrasound Approaches and the Role of UMAT in Enteral Nutrition Therapy

The increasing demand for usability and dynamic application of sonographic protocols in intensive care medicine is reflected in the development and implementation of approaches such as AGIUS, GUTS, and Lai. Their clearly defined measurement points and reproducible scoring systems provide structured longitudinal data but, because of their complexity and their reliance on static single measurements, may be of only limited dynamic use in daily clinical practice. Particularly in the context of enteral nutrition, there is a growing need for flexible, easily adaptable, and course-oriented methods.

Against this background, the Ultrasound Meal Accommodation Test (UMAT) is of increasing interest. It has demonstrated clinically relevant diagnostic performance for detecting feeding intolerance, with a 60-min change cutoff of 52% yielding a sensitivity of 50%, a specificity of 88.9%, a positive likelihood ratio of 4.50, and a negative likelihood ratio of 0.56 [9]. UMAT extends conventional, predominantly static ultrasound by adding a dynamic temporal component: it assesses the physiological adaptation and emptying capacity of the stomach after standardized nutrient intake by serial measurements of antral cross-sectional area (CSA) and gastric volume. The standardized protocol begins with a baseline fasting measurement, followed by ingestion of a defined test meal and subsequent ultrasound examinations at fixed time points. These serial measurements generate an individualized functional profile of gastric performance by documenting CSA and volume increase in the early postprandial phase and subsequent emptying, thereby providing a direct assessment of feeding tolerance.

Integration of UMAT into a pragmatic diagnostic algorithm introduces an additional, patient-centered dimension to enteral nutrition therapy:

- It may help objectify functional gastric adaptation and support earlier detection of pathological courses such as gastroparesis or motility disorders.
- In combination with baseline parameters from structured protocols (e.g., gastric CSA, small-bowel motility, and free intra-abdominal fluid), nutritional therapy may be tailored more closely to the individual patient, risks may be minimized, and complications may be identified at an earlier stage.
- UMAT is non-invasive, bedside-feasible, and flexible enough to be combined with different protocol approaches, resulting in a hybrid diagnostic framework that helps bridge scientific evidence and clinical practicality.

In summary, dynamic visualization of gastric accommodation and emptying by UMAT represents a potentially valuable extension of individualized, safe, and effective nutritional therapy in critically ill patients. By complementing the strengths of classical structured ultrasound protocols with a pragmatic longitudinal component, UMAT may contribute to a more nuanced management of gastrointestinal function in the intensive care setting.

## 4. Dynamic GIUS-Based Algorithms for Enteral Nutrition

Taken together, these considerations provide a strong rationale for simple and reproducible GIUS-based algorithms that translate dynamic gastrointestinal assessment into concrete feeding decisions at the bedside. Against this background, we propose three complementary variants that differ according to the patient’s current feeding status, clinical stability, and estimated risk of gastrointestinal dysfunction.

The proposed variants are intended as pragmatic, literature-informed decision aids rather than as prospectively validated standards. They are designed for integration into daily ward rounds and may be performed by appropriately trained members of the ICU team. Sonographic findings should always be interpreted together with hemodynamic stability, abdominal examination, gastrointestinal symptoms, medication exposure, metabolic parameters, and the overall clinical course.

For standardized bedside application, gastric antral cross-sectional area (CSA) is assessed in the supine or semi-recumbent position, preferably with the head of the bed elevated by approximately 30°. Serial examinations should be performed in the same patient position, imaging plane, and temporal relationship to enteral nutrient administration.

A gastric antral CSA below 6 cm^2^ is proposed as a pragmatic favorable finding during supine assessment. A CSA of 6 cm^2^ or greater, or a relevant increase compared with a previous examination, should be regarded as an alert finding rather than as an independent indication to interrupt enteral nutrition. The evidence context and limitations of the proposed reference values are summarized in Table 5.

The three variants represent different levels of monitoring intensity:

- Variant 1 is intended for intensified monitoring of patients receiving enteral nutrition who have an increased risk of feeding intolerance or a clinically dynamic gastrointestinal condition.
- Variant 2 is intended to assess gastrointestinal readiness before initiation or re-initiation of enteral nutrition.
- Variant 3 is intended for once-daily monitoring of clinically stable patients receiving apparently tolerated enteral nutrition.

Patients may transition between variants according to their clinical course. New clinical or sonographic abnormalities during Variant 3 should prompt escalation to Variant 1. Following initiation or re-initiation of feeding according to Variant 2, patients at increased risk of intolerance may initially be monitored according to Variant 1.

UMAT is not considered a routine component of all three variants. It may be used as an optional second-line dynamic test in selected, hemodynamically stable patients when conventional clinical assessment and static or serial GIUS findings remain inconclusive.

### 4.1. Shared Measurement Points and Standardized Acquisition

All three variants use a common set of sonographic measurement domains. For standardized bedside application, gastric antral cross-sectional area (CSA) should be assessed in the supine or semi-recumbent position, preferably with the head of the bed elevated by approximately 30°. Serial examinations should be performed in the same patient position, imaging plane, and temporal relationship to enteral nutrient administration.

The following findings are defined as core parameters:

- Gastric antral CSA and temporal trend:
  A CSA below 6 cm^2^ is regarded as a pragmatic favorable finding during standardized supine assessment. A CSA of 6 cm^2^ or greater, a relevant increase compared with the preceding examination, or persistent antral enlargement is regarded as an alert finding.
- Small-bowel diameter and temporal trend:
  A diameter below 3 cm without progressive dilatation is considered favorable. A diameter of 3 cm or greater, or increasing bowel dilatation on serial examination, represents an alert finding.
- Small- and large-bowel motility:
  Preserved intestinal motility is considered favorable. Markedly reduced or absent motility represents an alert finding. Where motility is quantified, the examined bowel segment and number of contractions per minute should be documented.

Additional findings are considered contextual parameters:

- Bowel wall or mucosal abnormalities;
- Free intra-abdominal or interloop fluid;
- Colonic wall abnormalities;
- Doppler-derived perfusion findings, where assessed.

Contextual parameters may strengthen the interpretation of the core findings but should not independently determine whether enteral nutrition is initiated, advanced, reduced, or interrupted. The evidence sources, population context, and limitations of the proposed reference values are summarized in Table 5.

### 4.2. Shared Decision Tree

The following decision criteria apply across all three variants:

- No abnormal core parameter:
  If gastric antral CSA is favorable, no relevant bowel dilatation is present, intestinal motility is preserved, and no clinical contraindication exists, enteral nutrition may be initiated, continued, or advanced according to the patient’s nutritional target and current guideline recommendations.
- One isolated abnormal core parameter:
  An isolated abnormal core parameter should not automatically lead to interruption of enteral nutrition. The clinical context, temporal trend, medication exposure, electrolyte status, and potentially reversible contributing factors should be reviewed. Enteral nutrition may be continued or initiated cautiously, and reassessment should be performed according to the selected clinical variant.
- Two or more concordant abnormal core parameters:
  If at least two core parameters concordantly indicate impaired gastrointestinal function, enteral nutrition should not be further advanced. In patients not yet receiving enteral nutrition, low-dose or trophic feeding may be initiated, provided that no contraindication or red-flag finding is present. In patients already receiving enteral nutrition, the feeding rate should be maintained at or reduced to a trophic level. Clinical and GIUS reassessment should be performed after 8 h before further advancement.

The numerical rule based on two or more concordant abnormal core parameters represents a pragmatic and hypothesis-generating component of the proposed framework. It has not yet been prospectively validated and should always be applied in conjunction with the overall clinical assessment.

#### Clinical and Sonographic Red Flags

Clinical or sonographic findings suggesting acute gastrointestinal pathology override the shared decision tree, irrespective of the number of abnormal core parameters.

Relevant sonographic red flags include:

- A mechanical obstruction pattern with marked or progressive bowel dilatation, pendular or “to-and-fro” movement, or a suspected transition point;
- Pneumatosis intestinalis, portal venous gas, or free intraperitoneal gas;
- Absent bowel wall perfusion, particularly when accompanied by absent motility or relevant bowel wall abnormalities;
- Rapidly progressive bowel dilatation with absent motility and increasing or complex free fluid.

Clinical red flags include persistent vomiting or regurgitation, rapidly progressive abdominal distension, signs of peritonitis, uncontrolled shock, or suspected abdominal compartment syndrome.

Red-flag findings are not included in the numerical count of core parameters. A single relevant red flag is sufficient to override the two-parameter rule. In these situations, enteral nutrition should be withheld or individually reconsidered, and established diagnostic and therapeutic pathways should be initiated.

### 4.3. Variant 1: Monitoring Ongoing Enteral Nutrition (Every 8 h) [Table 5]

Variant 1 is intended for patients already receiving enteral nutrition who have an increased risk of feeding intolerance or newly developing gastrointestinal dysfunction. Potential indications include recent feeding intolerance, increasing gastric retention, abdominal distension, impaired bowel motility, recent re-initiation of enteral nutrition, or abnormal findings during once-daily assessment.

GIUS is repeated every 8 h for a limited period until the clinical and sonographic findings have stabilized. The assessment focuses on gastric antral CSA and its temporal trend, small-bowel diameter, small- and large-bowel motility, and relevant changes in gastrointestinal morphology.

Gastric antral CSA, small-bowel diameter, and bowel motility are considered core parameters. Bowel wall or mucosal changes, free intra-abdominal fluid, colonic wall thickness, and Doppler-derived perfusion parameters may provide additional contextual information but should not be weighted equally or used as independent feeding stop criteria.

Once gastrointestinal function and feeding tolerance have stabilized, monitoring may be de-escalated to Variant 3.

### 4.4. Variant 2: Assessing Readiness to Initiate Enteral Nutrition [Table 5]

Variant 2 is intended to support the initiation or re-initiation of enteral nutrition after prolonged fasting, a clinically relevant interruption of feeding, paralytic ileus, gastrointestinal dysfunction, or stabilization following severe critical illness.

It is not intended to assess swallowing safety and should not replace dysphagia screening or instrumental swallowing assessment before oral nutrition.

The examination is performed before enteral nutrient administration and focuses on gastric filling, bowel dilatation, and gastrointestinal motility. The objective is to identify a sonographic pattern that supports the initiation of feeding and to detect findings that warrant a cautious approach or further evaluation.

Patients at increased risk of feeding intolerance should transition to Variant 1 once enteral nutrition has been initiated. Clinically stable patients with good tolerance may subsequently transition to Variant 3.

### 4.5. Variant 3: Once-Daily Assessment for Ongoing Enteral Nutrition [Table 5]

Variant 3 provides a pragmatic once-daily GIUS assessment for clinically stable patients receiving ongoing and apparently tolerated enteral nutrition. Its purpose is to support consistent evaluation of gastrointestinal function and to identify emerging abnormalities before overt feeding intolerance develops.

The examination is preferably performed at a standardized time each day and under comparable conditions. It focuses primarily on trends rather than on isolated measurements.

This once-daily approach facilitates workflow integration while preserving the option of immediate escalation to more intensive monitoring when the gastrointestinal or clinical condition changes.

### 4.6. Role of UMAT Within the Proposed Framework

UMAT is not considered a routine component of all three variants. In contrast to the static and serial parameters used in the standard algorithms, UMAT represents an optional dynamic functional test based on the gastric response to a standardized nutrient stimulus.

Within the proposed framework, UMAT may be considered as a second-line assessment in selected, hemodynamically stable patients when conventional clinical evaluation and static or serial GIUS findings remain inconclusive.

UMAT should not be performed when enteral nutrient administration is contraindicated, in patients with uncontrolled aspiration risk, suspected mechanical obstruction, severe gastrointestinal dysfunction, or ongoing hemodynamic instability. Its findings should be interpreted as complementary functional information and not as an independent criterion for initiating or withholding enteral nutrition.

### 4.7. Cutoff Values (Literature-Based [1,3,10,11,44,49])

**Figure 2:**
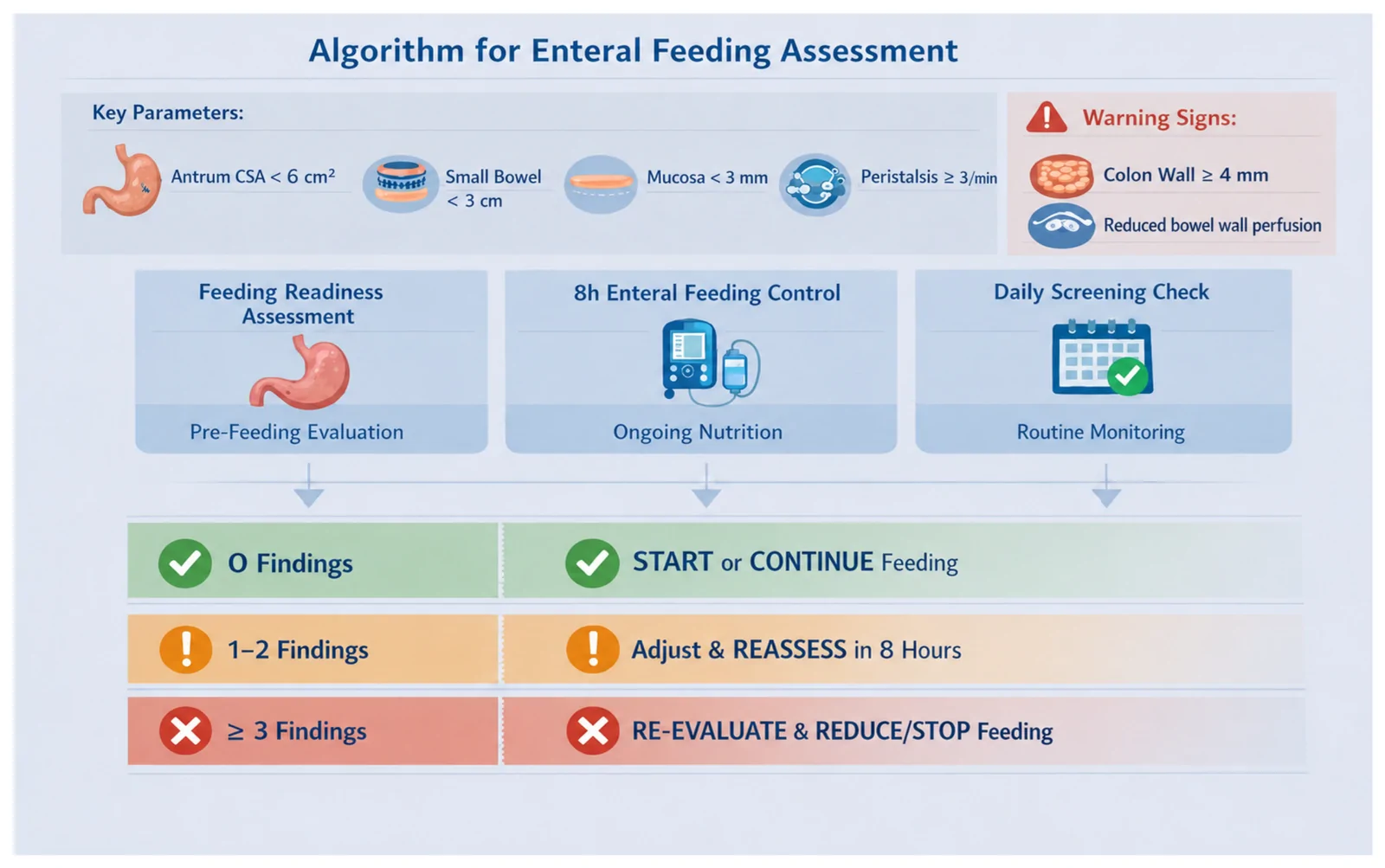
Schematic visualization of the proposed GIUS-based algorithm for enteral feeding assessment.

The proposed values are operational, literature-informed reference values and not prospectively validated standalone treatment thresholds. Patient position, imaging plane, timing relative to enteral nutrient administration, and serial trends should be documented. Measurements obtained in the supine and right lateral decubitus positions should not be used interchangeably (Table 5, Figure 2).

To illustrate the practical application of the proposed GIUS based monitoring algorithm, a representative clinical case study is presented in Appendix A.

## 5. Discussion

This review establishes a pragmatic, structured approach to sonographic monitoring of enteral nutrition in critically ill patients by synthesizing three protocol variants—regular 8 h assessments, preprandial readiness checks, and a once-daily standardized protocol—each defined by explicit, literature-based cutoff values for core GIUS parameters [1,7,10,49]. By operationalizing these measures, our framework aims to improve transparency and reproducibility and to support timely, individualized feeding decisions in intensive care practice [50].

The proposed GIUS framework is intended to complement rather than replace current ESPEN recommendations for enteral nutrition in critically ill patients. ESPEN recommends early enteral nutrition within 48 h when oral intake is not possible, followed by progressive advancement while avoiding early full feeding. Enteral nutrition should be delayed in the presence of established contraindications, including uncontrolled shock, overt bowel ischemia, or abdominal compartment syndrome [51].

The proposed GIUS algorithms are applied only after these clinical prerequisites and contraindications have been considered. In the absence of red flags, favorable GIUS findings support guideline-based initiation or advancement of enteral nutrition. Two or more concordant abnormal core parameters support a cautious trophic feeding strategy followed by clinical and sonographic reassessment after 8 h. This approach is consistent with the ESPEN principle of progressive enteral feeding but represents a hypothesis-generating extension, as current ESPEN guidelines do not provide GIUS-based decision thresholds.

Nevertheless, important unresolved questions underlie our protocol selection and the proposed measurement intervals. Probabilities of intolerance detection vary widely across patient populations, and the predictive accuracy of specific GIUS markers under routine clinical conditions remains incompletely validated. While elements such as the gastric antral CSA and small-bowel diameter are widely used, the reliability of additional markers—including mucosal thickness, colonic wall measurements, and Doppler indices—has yet to be substantiated by large-scale prospective trials. Much of the published evidence consists of expert consensus or retrospective data and lacks robust linkage to patient-centered outcomes, which limits generalizability and risk stratification [41,42,52,53,54,55,56,57,58].

Against this background, our structured protocol may provide a useful foundation for clinical monitoring, but the evidence gaps emphasize the need for ongoing research and multicenter collaboration to refine sonographic algorithms and clarify their impact on patient outcomes. As enteral nutrition protocols evolve, future work should validate measurement intervals, optimize parameter selection, and integrate GIUS findings with additional biomarkers for a more comprehensive assessment.

A major strength of our algorithmic approach lies in the explicit operationalization of cutoff values, which may facilitate reproducibility and interdisciplinary communication. The structured use of GIUS provides an immediate bedside assessment tool and may help clinicians react more promptly to evolving gastrointestinal conditions, thereby reducing the risks of underfeeding, aspiration, or unnecessary interruptions. By defining distinct time intervals—ranging from frequent (8-hourly) to pragmatic daily schedules—the protocol also offers flexibility that can be adapted to patient acuity, resource availability, and institutional workflow.

However, these potential advantages must be weighed against challenges inherent to GIUS methodology in the ICU setting. Not all parameters are supported by the same strength of evidence [11,44]. While the gastric antral CSA and small-bowel diameter have been more frequently validated as markers of gastric emptying and small-bowel dilatation, other variables—such as mucosal thickness, colonic wall thickness, and resistive index—are supported by more limited data and may be more susceptible to inter-operator variability [58,59]. Furthermore, direct correlations between these sonographic measurements and clinically meaningful outcomes (e.g., incidence of pneumonia, successful advancement of enteral nutrition, ICU length of stay) have not yet been robustly demonstrated in randomized multicenter trials [3].

An additional methodological limitation concerns patient positioning during gastric ultrasound. Many perioperative and diagnostic studies derive antral CSA cutoffs from measurements obtained in the right lateral decubitus position to optimize antral filling and visualization. In contrast, our proposed cutoff values are based on supine measurements, following the examination conditions described by Lai et al., which better reflect routine practice in critically ill patients who are predominantly managed in the supine position. Although supplementary assessment in the right lateral decubitus position may enhance image quality in selected cases, such positional changes are not always feasible in the ICU and may limit direct transferability of right lateral-derived thresholds to critically ill populations.

Clinical implementation also encounters practical barriers. Variability in training, availability of equipment, and differences in patient anatomy can affect feasibility and reproducibility [52,53,60,61,62,63]. In addition, the relevance of individual parameters may vary in patients with comorbidities (e.g., chronic gastrointestinal disease, obesity, prior abdominal surgery) or under the influence of medications that affect motility or mucosal integrity [52,64,65].

Successful implementation of the proposed GIUS framework requires structured training and competency assessment. EFSUMB recommends dedicated training in bowel ultrasound, preferably following education in general abdominal ultrasound, and emphasizes standardized examination, supervision, documentation, and quality assurance [66]. The extensive examination numbers proposed in earlier EFSUMB curricula refer to comprehensive gastroenterological ultrasound and should not be directly transferred to a focused ICU-GIUS protocol.

Evidence from focused ultrasound applications indicates that clearly defined sonographic tasks can be acquired within a substantially shorter training pathway. For qualitative gastric ultrasound, structured theoretical and practical training followed by approximately 24–33 supervised examinations was associated with 90–95% procedural success [63,67,68,69]. In addition, focused gastric ultrasound has been performed successfully by ICU nurses following a short formal training program, with good agreement between nurse-performed ultrasound estimates of gastric residual volume and conventional assessment [68]. Studies involving medical students further support the feasibility of teaching standardized focused ultrasound tasks through brief theoretical instruction followed by supervised practical examinations [63,67,68,69].

Based on these findings, we propose a competency-based rather than profession-based training model. Physicians and other appropriately trained ICU professionals, including nurses where permitted by local governance, may perform standardized image acquisition and measurements after completing theoretical instruction, hands-on training, and a documented series of supervised examinations. Competency should be confirmed by direct assessment of image acquisition, anatomical identification, measurement accuracy, interpretation of core parameters, and recognition of findings requiring escalation.

A practical training pathway may include theoretical education, supervised hands-on scanning, a logbook of approximately 20–30 focused examinations, and formal assessment before independent protocol-based acquisition. This proposed number represents a pragmatic starting point derived mainly from gastric ultrasound learning-curve studies and should not be regarded as a validated universal competency threshold [63,70].

Comprehensive interpretation of complex findings, including suspected obstruction, bowel ischemia, pneumatosis, or impaired bowel wall perfusion, requires advanced GIUS experience and should prompt review by an appropriately experienced physician or sonographer. Final decisions regarding initiation, advancement, reduction, or interruption of enteral nutrition remain the responsibility of the treating multidisciplinary team.

Nevertheless, the structured daily protocol and its variable-frequency alternatives represent a promising step toward more harmonized practice, support standard operating procedures, and provide a reproducible basis for further innovation [61]. Continued outcome-driven research and collaborative multicenter trials are necessary to validate parameter selection, optimize cutoff values, and confirm the clinical benefits of GIUS protocols for enteral feeding monitoring in the ICU context [6].

Taken together, these findings underscore both the promise and the responsibility associated with structured GIUS protocols for enteral nutrition management in the ICU. While this approach promotes objectivity, workflow integration, and standardization, the utility of individual parameters and measurement intervals remains dependent on context, expertise, and evolving evidence. Current algorithmic frameworks provide a useful foundation for practice and research, yet the literature clearly highlights the need for large-scale validation, harmonized training, and outcome-focused studies. Until then, interdisciplinary collaboration and critical reappraisal remain essential to ensure that GIUS-based protocols enhance care without overreliance and that each decision is integrated with comprehensive clinical assessment.

## 6. Conclusions

Gastrointestinal ultrasound offers a feasible and non-invasive bedside method to support personalized enteral nutrition therapy in critically ill patients. When applied within a structured protocol, GIUS may help clinicians assess feeding readiness, monitor gastrointestinal tolerance, and identify early signs of gastrointestinal dysfunction. Rather than replacing clinical judgment, GIUS should be integrated into a multimodal decision-making process including hemodynamic stability, metabolic markers, abdominal findings, and the overall clinical course.

The proposed algorithm provides a pragmatic framework for translating GIUS findings into bedside nutritional decisions. However, the suggested thresholds and decision pathways require prospective validation before routine implementation can be recommended. Future studies should evaluate standardization, reproducibility, and the effect of GIUS-guided nutrition strategies on feeding tolerance, caloric target achievement, and patient-centered outcomes.

## Figures and Tables

**Table 1:** The GUTS Protocol.

Segment	Parameters/ Measurements	Pathological Cutoffs	Ancillary Diagnostics	Remarks
Stomach	Antral CSA, wall thickness, motility, residual volume	Wall thickness > 3 mm; diameter > 25 mm; residual volume > 500 mL fasting	—	Assessment of filling state
Small intestine	Wall thickness, luminal diameter, peristalsis	Wall thickness > 3 mm; diameter > 25 mm; hypomotility < 3/min	—	Detection of inflammation, dilatation, ischemia
Colon	Wall thickness, luminal diameter, motility	Wall thickness > 4 mm; diameter > 60 mm; hypomotility	—	Identification of megacolon and inflammatory changes
Ancillary	Free fluid, pneumatosis, Doppler perfusion	RI > 1.0–1.2; free fluid > 1 cm; pneumatosis	Doppler perfusion assessment	Complications including ischemia and sepsis

**Table 2:** The AGIUS Protocol.

Segment	Parameters/ Measurements	Scoring (0–3)	Thresholds (Physiological/Pathological)	Remarks
Stomach	Wall thickness, motility, filling state	0–3 depending on findings	Wall thickness < 3 mm; peristalsis ≥ 3/min	Includes antral contraction and residual volume
Small intestine	Wall thickness, diameter, peristalsis, free fluid	0–3 depending on findings	Wall thickness < 3 mm; diameter < 25 mm; peristalsis ≥ 3/min	Detection of free fluid
Colon	Wall thickness, diameter, motility, stratification	0–3 depending on findings	Wall thickness < 4 mm; diameter < 60 mm	Assessment for inflammation and ileus
Free fluid	Quantification	0–3 depending on volume	—	Evaluation for peritonitis and edema

**Table 3:** The Lai. Protocol.

Domain	Parameter and Acquisition	Study-Derived Value Associated with Successful EN	Clinical Role	Important Limitation
Stomach	Gastric antral CSA determined by tracing the antral contour using Simpson’s integral method	≤9 cm^2^	Assessment of gastric filling as part of the composite model	Primarily measured in RLD; supine permitted when RLD was not feasible; no position-specific analysis
Colon	Internal diameter of the left descending or right ascending colon	≤2.9 cm	Structural indicator of colonic distension or recovery	Colonic value; not applicable as a small-bowel threshold
Colonic motility	Peristaltic contractions in the examined colonic segment	>3/min	Functional indicator of colonic motility	Limited to the examined segment and operator-dependent
Composite assessment	Combination of antral CSA, colonic diameter, and colonic peristalsis	AUC 0.95	Supported the timing of EN initiation in patients with AGI II	Single-center retrospective derivation cohort without external validation

**Table 4:** Evidence-related GIUS parameters used in the proposed enteral feeding framework.

Parameter	Operational Reference	Role	Evidence	Key Limitations
**Gastric antral CSA**	<6 cm^2^ favorable; ≥6 cm^2^ or increasing alert	Core	Protocol/review [1]; ICU FI cutoff 6 cm^2^ [44]; CSA ≤ 9 cm^2^ in AGI-II model [49]	Position-, population-, and endpoint-dependent; values are not interchangeable
**Small-bowel diameter**	<3 cm favorable; ≥3 cm or progressive dilatation alert	Core	Protocols [1,10]; prospective ICU study [11]	Structural marker; limited validation for feeding decisions
**Intestinal motility**	Preserved favorable; markedly reduced or absent alert	Core	Protocols [1,10]; small-bowel motility [11]; colonic motility >3/min [49]	Segment- and operator-dependent; thresholds not interchangeable
**Colonic diameter**	≤2.9 cm in the Lai model	Context-specific core component	Composite EN-initiation model in AGI II [49]	Single-center derivation; not externally validated
**Bowel wall/mucosal morphology**	No fixed cutoff; assess thickness, folds, and stratification	Contextual	Protocols [1,10]; prospective ICU findings [11]	Segment-, disease-, and technique-dependent
**Free abdominal/interloop fluid**	New, increasing, or complex fluid as warning finding	Contextual	Structured GIUS assessment [1,10]; GI dysfunction framework [3]	Non-specific; influenced by ascites, fluid balance, inflammation, and surgery
**Bowel wall perfusion**	Preserved vs. markedly reduced or absent	Contextual/red flag	Protocols [1,10]; GI dysfunction framework [3]	Doppler-, operator-, and hemodynamic-dependent; no validated RI cutoff
**Acute pathological pattern**	Obstruction pattern, pneumatosis, portal venous or free gas, absent perfusion	Red flag	Structured GIUS and GI dysfunction literature [1,3,10]	Requires confirmatory diagnostics; not included in the numerical count

**Table 5:** Clinical application and limitations of the three proposed GIUS variants.

Feature	Variant 1: 8 h Monitoring	Variant 2: Feeding Readiness	Variant 3: Once-Daily Monitoring	Key Limitation
**Target population**	Ongoing EN with suspected intolerance or dynamic GI dysfunction	Before initiation or re-initiation of EN	Stable patients with apparently tolerated EN	Clinical indications require prospective validation
**Typical indication**	Recent intolerance, abnormal GIUS, distension, reduced motility, or recent restart of EN	After fasting, feeding interruption, ileus, GI dysfunction, or stabilization	Routine monitoring during stable EN	Overlap between variants may occur
**Timing**	Every 8 h until stabilization	Once before EN initiation	Once daily under comparable conditions	Optimal monitoring intervals are not validated
**Main objective**	Detect short-term changes and guide adjustment of ongoing EN	Assess GI readiness for cautious EN initiation	Detect emerging dysfunction before overt intolerance	GIUS supplements, but does not replace, clinical assessment
**Measurement focus**	Serial changes in core parameters	Baseline core parameters before feeding	Daily trends in core parameters	Acquisition must be standardized
**No abnormal core parameter**	Continue or advance EN	Initiate and progressively advance EN	Continue or advance EN	Guideline-based clinical criteria still apply
**One abnormal core parameter**	Continue cautiously; reassess after 8 h	Start cautiously at reduced or trophic rate; reassess early	Continue with clinical review; repeat within 24 h	Single abnormalities may be nonspecific
**≥2 concordant abnormal core parameters**	Do not advance; maintain or reduce to trophic EN; reassess after 8 h	Initiate only trophic EN if no contraindication; reassess after 8 h	Do not advance; maintain or reduce to trophic EN; switch to Variant 1	Numerical rule is pragmatic and not prospectively validated
**Red-flag finding**	Override algorithm; initiate established diagnostic pathway	Do not initiate routine EN through the algorithm	Immediate reassessment independent of schedule	A single red flag overrides the parameter count
**Transition**	De-escalate to Variant 3 after stabilization	Proceed to Variant 1 if high risk; otherwise to Variant 3 after stable tolerance	Escalate to Variant 1 if abnormalities develop	Transition criteria remain clinically determined

**Figure A1:**
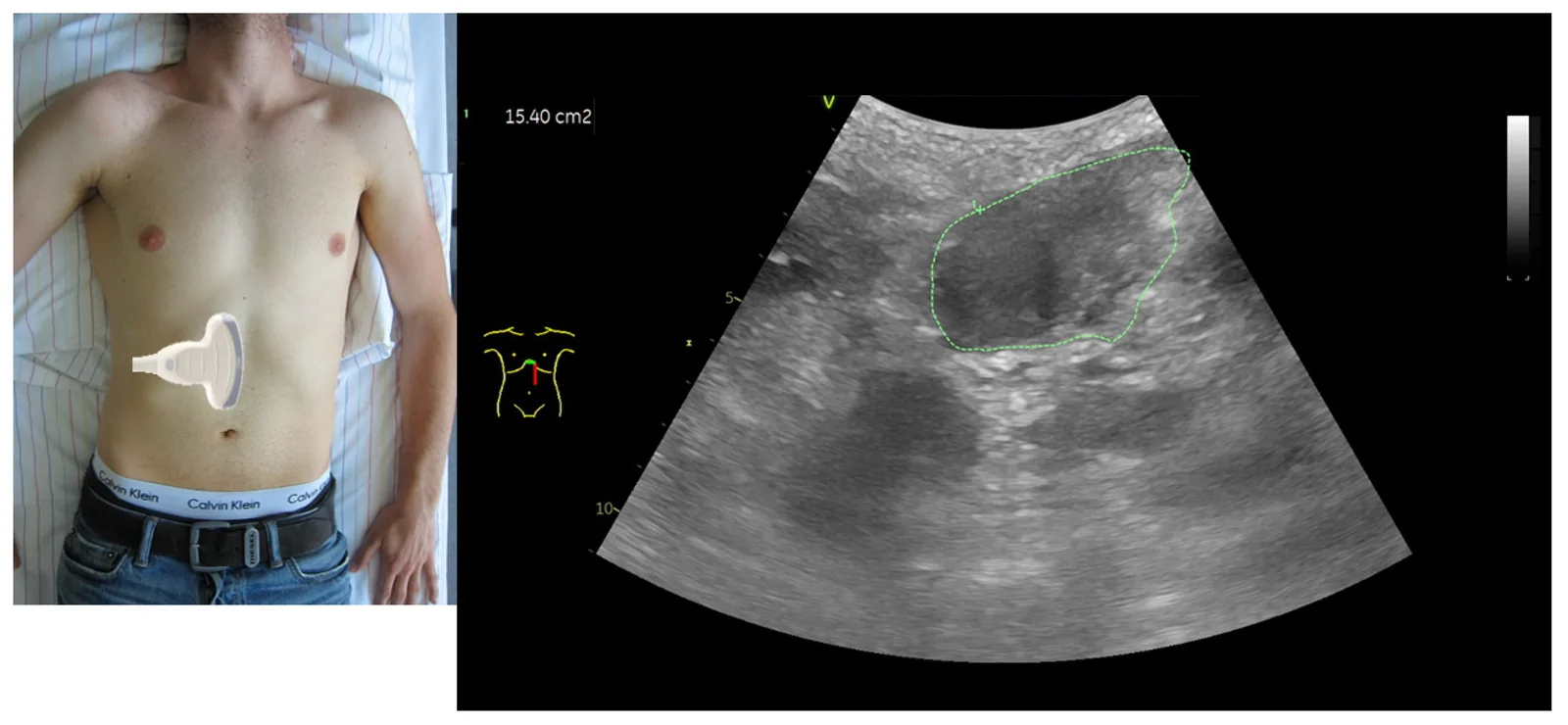
Representative gastric ultrasound finding from the illustrative case. The green dashed contour outlines the gastric antrum. The measured antral cross-sectional area (CSA) of 15.4 cm^2^ is consistent with increased gastric retention.

**Figure A2:**
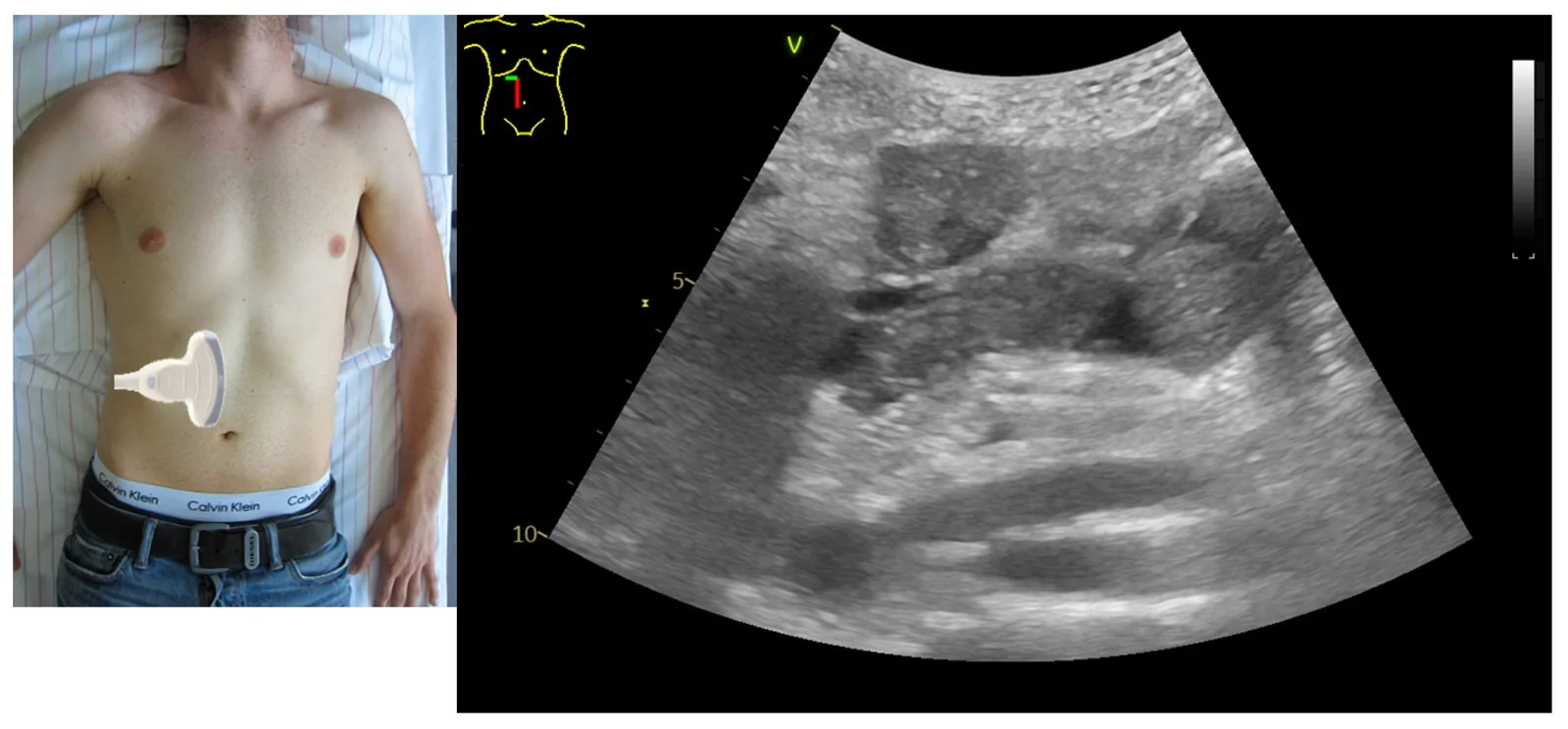
Representative intestinal ultrasound views from the illustrative case. Upper panel: mildly distended bowel loop. Lower panel: free intraperitoneal fluid between bowel loops.

**Figure A3:**
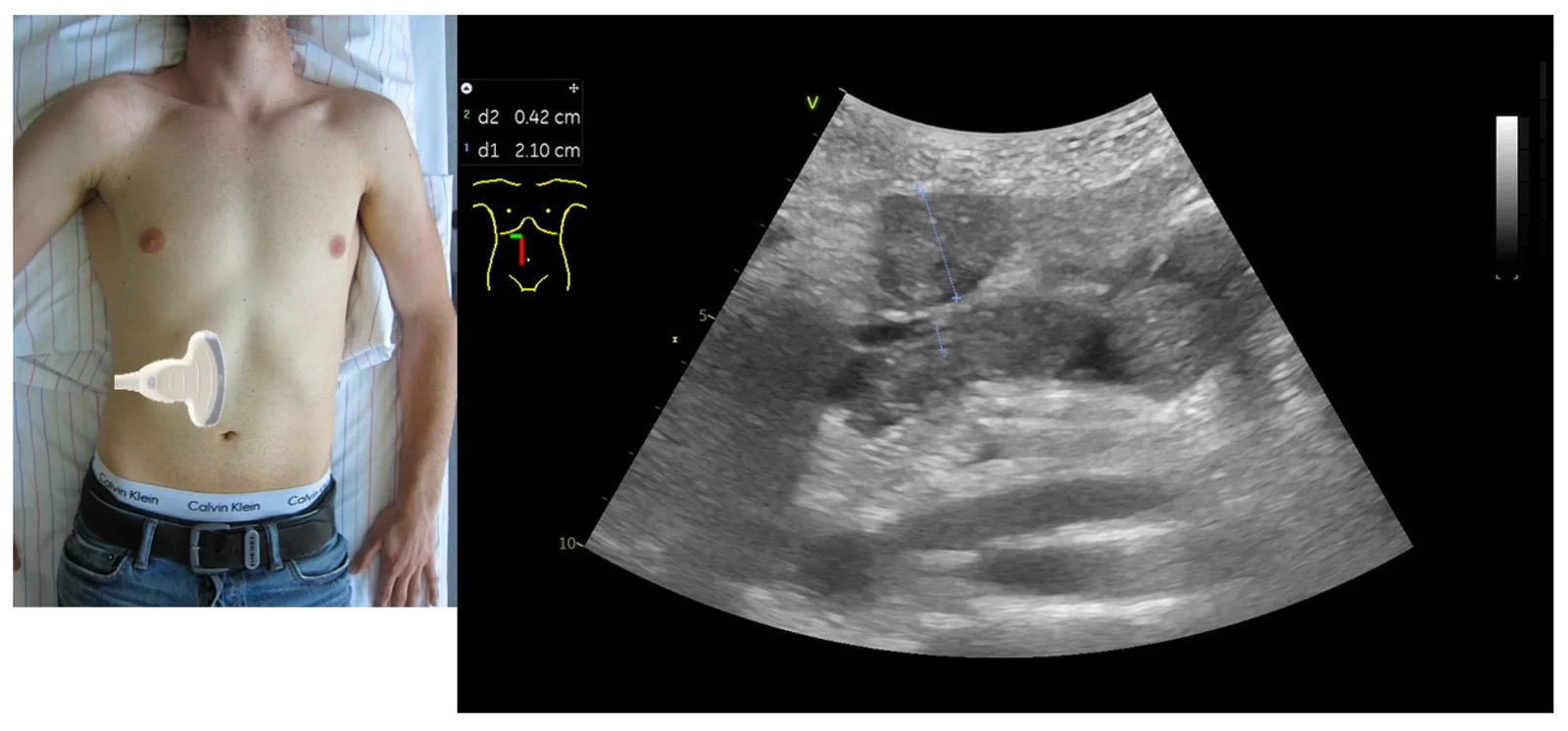
Representative small-bowel ultrasound image. d1 indicates a small-bowel diameter within the normal range (2.1 cm), whereas d2 indicates increased small-bowel wall thickness.

**Figure A4:**
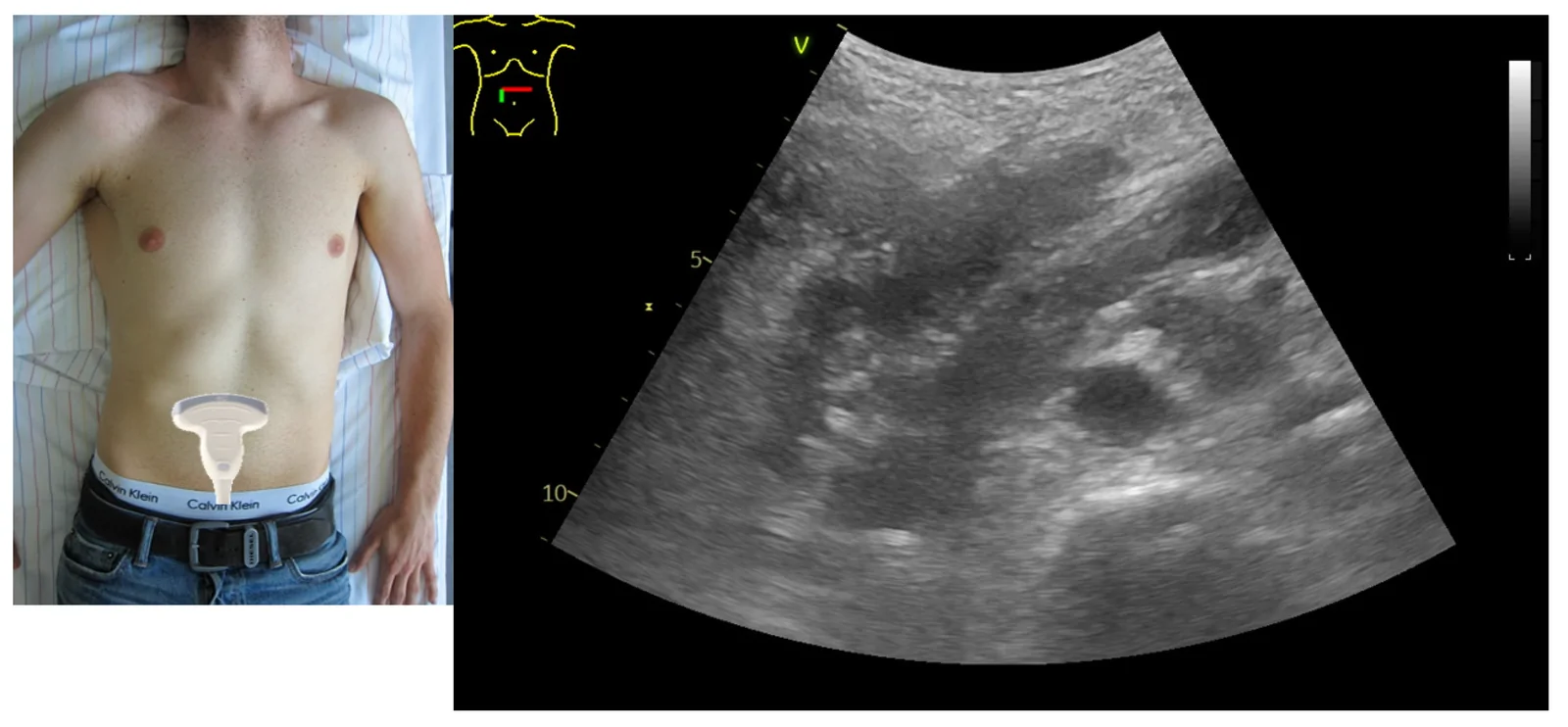
Transverse intestinal ultrasound view demonstrating variable small-bowel diameters. In dynamic real-time assessment, differences in luminal diameter should be interpreted together with bowel wall motion and peristaltic activity. In this example, the varying diameters are consistent with preserved peristalsis.

## Data Availability

No new data were created or analyzed in this study. Data sharing is not applicable to this article.

## Abbreviations

The following abbreviations are used in this manuscript:

Abbreviation	Full term
AGIUS	Acute Gastrointestinal Injury Ultrasound Score
AGI	Acute Gastrointestinal Injury
AUC	Area under the curve
BMI	Body Mass Index
CSA	Cross-sectional area
CT	Computed Tomography
EN	Enteral nutrition
ESPEN	European Society for Clinical Nutrition and Metabolism
GI	Gastrointestinal
GIUS	Gastrointestinal ultrasound
GRV	Gastric residual volume
GUTS	Gastrointestinal and Urinary Tract Sonography
ICU	Intensive care unit
I-FEED	Intolerance of feeding, food intolerance, examination, enteral nutrition, duration of symptoms
NPV	Negative Predictive Value
POCUS	Point-of-Care Ultrasound
PN	Parenteral nutrition
PPV	Positive Predictive Value
RI	Resistive index
ROC	Receiver operating characteristic
SCCM	Society of Critical Care Medicine
SD	Standard deviation
SOFA	Sequential Organ Failure Assessment
UMAT	Ultrasound Meal Accommodation Test
VExUS	Venous Excess Ultrasound

## Appendix A. Patient Example

In this section, we present a representative case to illustrate how the proposed algorithm may support bedside nutritional decision-making. The patient had pneumonia complicated by prolonged shock and initially required vasopressor support. GIUS assessment was performed after relative hemodynamic stabilization had been achieved and vasopressor therapy was already being stepwise de-escalated. Ultrasound examination was performed in the supine position and included standardized assessment of the gastric antrum and bowel, with the corresponding probe positions shown alongside the ultrasound images.

GIUS demonstrated marked gastric retention, with an antral cross-sectional area (CSA) of approximately 15.4 cm^2^ (Figure A1), consistent with impaired gastric emptying. In addition, bowel ultrasound showed mildly distended and edematous small-bowel loops, with free intraperitoneal fluid visible between adjacent bowel loops (Figure A2). Quantitative assessment of the bowel further demonstrated a small-bowel diameter within the non-dilated range (d1 = 2.1 cm), while bowel wall thickness was increased (d2 = 0.42 cm), consistent with intestinal wall edema (Figure A2). Dynamic real-time assessment revealed variable luminal diameters in transverse view, supporting the presence of preserved, although reduced, peristaltic activity rather than complete paralytic ileus (Figure A3 and Figure A4).

Taken together, these findings suggest relevant gastrointestinal dysfunction with impaired gastric emptying, bowel wall edema, and associated free intraperitoneal fluid, while residual intestinal motility appears to be preserved. Within the framework of the proposed algorithm, this pattern would favor reduction rather than complete cessation of enteral nutrition, combined with close reassessment and consideration of supplemental parenteral nutrition if caloric targets cannot be achieved.
